# Incorporating quality assessments of primary studies in the conclusions of diagnostic accuracy reviews: a cross-sectional study

**DOI:** 10.1186/1471-2288-14-33

**Published:** 2014-03-03

**Authors:** Eleanor A Ochodo, Wynanda A van Enst, Christiana A Naaktgeboren, Joris AH de Groot, Lotty Hooft, Karel GM Moons, Johannes B Reitsma, Patrick M Bossuyt, Mariska MG Leeflang

**Affiliations:** 1 Department of Clinical Epidemiology, Biostatistics & Bioinformatics, Academic Medical Center, University of Amsterdam, Meibergdreef 9, 1105 AZ Amsterdam, The Netherlands; 2 Dutch Cochrane Centre, Academic Medical Center, Amsterdam 1105 AZ, The Netherlands; 3 Julius Center for Health Sciences and Primary Care, University Medical Center Utrecht, 3508 GA Utrecht, The Netherlands; 4 Centre for Evidence-based Health Care, Faculty of Medicine & Health Sciences, Stellenbosch University, Cape Town, South Africa

**Keywords:** Diagnostic tests, Test accuracy, Systematic reviews, Meta-analysis, Quality, QUADAS, Risk of bias

## Abstract

**Background:**

Drawing conclusions from systematic reviews of test accuracy studies without considering the methodological quality (risk of bias) of included studies may lead to unwarranted optimism about the value of the test(s) under study. We sought to identify to what extent the results of quality assessment of included studies are incorporated in the conclusions of diagnostic accuracy reviews.

**Methods:**

We searched MEDLINE and EMBASE for test accuracy reviews published between May and September 2012. We examined the abstracts and main texts of these reviews to see whether and how the results of quality assessment were linked to the accuracy estimates when drawing conclusions.

**Results:**

We included 65 reviews of which 53 contained a meta-analysis. Sixty articles (92%) had formally assessed the methodological quality of included studies, most often using the original QUADAS tool (n = 44, 68%). Quality assessment was mentioned in 28 abstracts (43%); with a majority (n = 21) mentioning it in the methods section. In only 5 abstracts (8%) were results of quality assessment incorporated in the conclusions. Thirteen reviews (20%) presented results of quality assessment in the main text only, without further discussion. Forty-seven reviews (72%) discussed results of quality assessment; the most frequent form was as limitations in assessing quality (n = 28). Only 6 reviews (9%) further linked the results of quality assessment to their conclusions, 3 of which did not conduct a meta-analysis due to limitations in the quality of included studies. In the reviews with a meta-analysis, 19 (36%) incorporated quality in the analysis. Eight reported significant effects of quality on the pooled estimates; in none of them these effects were factored in the conclusions.

**Conclusion:**

While almost all recent diagnostic accuracy reviews evaluate the quality of included studies, very few consider results of quality assessment when drawing conclusions. The practice of reporting systematic reviews of test accuracy should improve if readers not only want to be informed about the limitations in the available evidence, but also on the associated implications for the performance of the evaluated tests.

## Background

Systematic reviews of diagnostic test accuracy form a fundamental part of evidence-based practice [1,2]. An essential part of a systematic review is the evaluation of the risk of bias [3] also referred to as assessment of methodological quality [4]. Limitations in the design and conduct of the study may lead to overestimation of the accuracy of the test under study [5,6]. This is of concern, because tests introduced in practice based on weak evidence may lead to misdiagnosis, improper management of patients and, subsequently, poor health outcomes [7-9]. Such limited evidence could also lead to unnecessary testing and avoidable health care costs [7].

The Quality Assessment for Diagnostic Accuracy Studies tool (QUADAS) has been developed and introduced to evaluate the methodological quality of studies included in systematic reviews of test accuracy [10]. A revised version, QUADAS-2, was introduced in 2011. The revised instrument considers methodological quality in terms of risk of bias and concerns regarding the applicability of findings to the research question. It does so in four key domains: patient selection, index test, reference test, and patient flow [11]. The QUADAS-2 tool is recommended by the U.K National Institute for Health and Clinical Excellence, the Cochrane Collaboration, and the U.S. Agency for Healthcare Research and Quality.

The use of QUADAS in test accuracy reviews to assess the methodological quality of included primary studies is increasing. Willis and Quigley reported that 40% of diagnostic reviews published between 2006 and 2008 used the QUADAS tool [12], while Dahabreh and colleagues reported that, in 2004, about 2% of diagnostic reviews used QUADAS, while 44% did so in 2009 [13].

Simply assessing quality without interpreting and using the results to draw conclusions is not sufficient in evidence synthesis. The results from quality assessment should be used to make inferences about the validity of the results.

The challenge of incorporating quality assessments of the included studies into the overall findings of a review is well known in intervention reviews. Moja and colleagues [14] reported that just about half of the 965 reviews they examined had incorporated the results of quality assessment in their analysis and interpretation of the results of their studies. A similar study done almost 10 years later by Hopewell and colleagues [15] reported that only 41% of the 200 reviews they examined incorporated the risk of bias assessment into the interpretation of their conclusions. The challenge of incorporating results of quality assessment in the conclusions may also be present in diagnostic accuracy reviews.

Readers, who usually have limited or basic knowledge of the methodological process involved in diagnostic reviews, often focus exclusively on the conclusion sections of a review when arriving at a judgment about a test’s performance [16]. In this regard, drawing conclusions without considering the risk of bias in included studies may lead to unwarranted optimism about the value of the test(s) under study. We sought to identify to what extent – and, if so, how – quality assessment is incorporated in the conclusions of diagnostic accuracy reviews.

## Methods

This study was part of a larger meta-epidemiological study to examine the methodology used in recent test accuracy reviews. Since diffusion of methods takes time, we focused on recently published reviews. On 12^th^ September 2012, we identified a convenience sample of test accuracy reviews indexed in the databases MEDLINE and EMBASE between 1^st^ May and 11^th^ September 2012 using the search strategy available in Additional file 1.

Eligible were reviews with a systematic search and methodology in appraising and summarising studies that evaluated a medical test against a reference standard. These reviews could present summary accuracy measures generated in a meta-analysis or present a range of accuracy measures without a summary measure. We included reviews published in English and which evaluated human studies dealing with patient data (as opposed to specimen data). We excluded individual patient data reviews and reviews evaluating the accuracy of prognostic tests in predicting future events. The methodology for evaluating quality in reviews of prognostic tests is less well developed than that for diagnostic tests.

The data extraction form was pilot tested by performing double data extraction on a third of the articles (by E.O., W.E., C.N., J.G., L.H., and M.L.). Discrepancies were discussed and unclear questions on the form were made more specific. Data extraction was then performed by one researcher (by E.O., W.E., C.N., and M.L.) using the standardized form and checked by another researcher (by E.O., W.E., C.N., and M.L.). Disagreements were resolved through discussion and when necessary by including a third reviewer (P.B.).

As conclusions are influenced largely by the methods used and the results produced in a review, we first examined every included review to check if methodological quality of included studies had been assessed using the recommended tool, QUADAS or QUADAS-2 [10,11], or any other tool that the authors specified as a system to assess risk of bias.

We examined the abstracts to check if methodological quality was mentioned in any of the sections (background, methods, results and conclusions). Abstracts are the most commonly read part of articles and readers often rely on abstracts to give them a snapshot of the content of reviews; where full texts cannot be accessed, judgments of a test’s performance may be made on abstracts alone [17-19].

We examined the main body of the review to check if the methodological quality of included studies was assessed, which tool had been used to assess quality, how results of quality assessments were presented, if the quality of studies had influenced the decision to perform a meta-analysis, if and how an assessment of quality was incorporated into the analysis, and if and how the results of quality assessment were discussed and eventually used in drawing conclusions about the test.

We regarded quality as being incorporated into the conclusions of the review when results of quality assessment of the included studies, or limitations surrounding quality assessment, were considered together with the accuracy estimates of the diagnostic tests in drawing conclusions about the performance of the test(s) under evaluation. We distinguished between drawing conclusions about test performance and making recommendations for future research. Conclusions of test performance are usually based solely on the results of the review and could be used as guidance for clinical practice, whereas recommendations for research are generally made after considering additional information not necessarily investigated in the review itself.

## Results

### Search results

**Figure 1:**
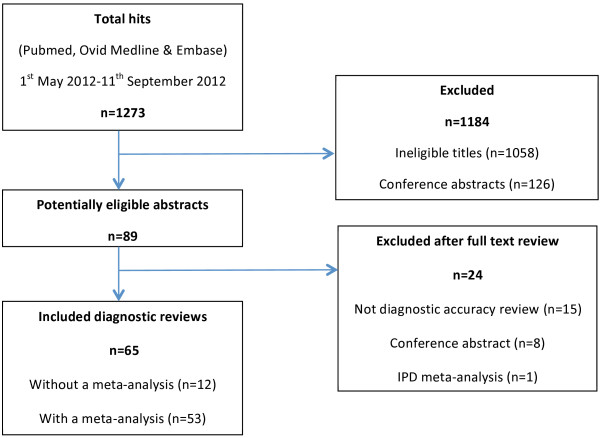
Flow chart of study inclusion.

The initial search identified 1,273 articles. We excluded 1,184 articles after screening titles and abstracts, and had to exclude 24 more articles after reading the full text. Sixty-five reviews were eventually included in this study on quality assessment. Of these reviews, 53 contained a meta-analysis (see Figure 1).

### Characteristics of included reviews

Details of the study characteristics are outlined in Table 1. In summary, this sample of 65 reviews included one Cochrane review and 64 reviews published in other peer-reviewed journals. The median impact factor of the journals in which the included reviews were published in was 3.1 [Interquartile range, 2.4 to 4.1]. Of all the tests evaluated in the included reviews, imaging tests formed the largest group (n = 36, 55%).

**Table 1:** Characteristics of included reviews

**Characteristic**		**Number (%) N = 65**
Number of primary studies in reviews, median [IQ range]		16 [10–24]
Journal impact factor, median [IQ range]		3.1 [2.4-4.1]
Type of test evaluated		
	Imaging test	36 (55)
	Laboratory test	17 (26)
	Other	12 (18)
Publication		
	Cochrane library	1 (1)
	Other peer reviewed journals	64 (99)
Quality assessment tools		
	No quality assessment	5 (8)
	QUADAS	44 (68)
	QUADAS-2	1 (1)
	STARD	3 (5)
	Both QUADAS and STARD	4 (6)
	Quality assessment of reliability studies	1 (1)
	Other checklists of quality criteria	6 (9)
	Unclear	1 (1)
Presentation of quality results*		
	Table of individual quality items	31 (48)
	Summary score	18 (28)
	Summary graph	12 (18)
	Narrative explanation	7 (11)
	Other	5 (8)

*One review could have one or more ways of presenting results.

### Instruments used to assess methodological quality

Of the included reviews, 60 (92%) had formally assessed the methodological quality of included studies. Most reviews had used QUADAS to assess the quality of included studies (n = 44) and most presented their results as tables of individual quality items (n = 31). Details of this assessment are outlined in Table 1.

### Incorporation of assessments of quality in the review

a. Abstract

Table 2 summarizes the approaches used to mention quality in the abstract of the review with examples. Quality assessment was only mentioned in 28 abstracts (43%); a majority of these referred to it in the methods section (n = 21). Only 5 reviews [20-24] linked results of quality assessment to accuracy estimates in the conclusion of the abstract. Three of these had not performed a meta-analysis [22-24], due to the poor quality of included studies.

b. Main text

**Table 2:** Incorporation of quality assessment in abstracts of diagnostic reviews

**Approach**	**Overall quality of included studies**	**Number N = 65**	**Example**
**Quality mentioned in abstract**		**28 (43%)**^**a**^	
Quality in methods		21 (32%)	The quality of the studies was assessed using the guidelines published by the QUADAS (quality assessment for studies of diagnostic accuracy, maximum score 14) [25].
Quality in results		12 (19%)	“The sensitivity analysis of 10 high quality studies (a score of > =4) showed a pooled sensitivity of 94% and pooled specificity of 0.95” [26]
			“The quality of the included studies was poor to mediocre” [27]*.*
Quality results considered in conclusion		5 (8%)	α“The observed high sensitivity of the punch biopsy derived from all studies is probably the result of verification bias” [20].
			β“The quality of the studies investigating these tests is too low to provide a conclusive recommendation for the clinician” [23].

^a^Quality was mentioned in one or more sections in the abstract.

α Example of conclusion in a review with a meta-analysis.

β Example of conclusion in a review without a meta-analysis.

Table 3 summarizes, with examples, the approaches used to incorporate quality in the main text of the review. The detailed breakdown of how quality was incorporated in the analysis, discussion and eventually to the conclusions in the main text of the review is presented below.

**Table 3:** Incorporation of quality assessment in main text of diagnostic reviews

**Approach**	**Overall quality of included studies**	**Number N = 65**	**Example**
**Quality mentioned in the main text**		**60 (92%)**^**b**^	
Results of quality assessment reported, no mention in discussion or conclusion		13 (20%)	Results presented as table of individual QUADAS items. No further discussion or interpretation of results [28].
Results of quality assessment reported and discussed, but quality not linked to conclusion		41 (63%)	Assessed quality using criteria of internal and external validity. Overall quality clearly not stated.
			Discussion as limitation only: “Fourth, the variability in the quality of the primary studies may introduce important limitations for the interpretation of this review study”.
			Conclusion: “Based on the results of this systematic review, F-18 FDG PET (PET/CT) was useful in ruling in extrahepatic metastases of HCC and valuable for ruling out the recurrent HCC” [29].
Results of quality assessment reported and discussed, and conclusions regarding test accuracy linked to results of quality assessment		6 (9%)	α“ In conclusion, the observed high sensitivity and low specificity of the colposcopy-directed punch biopsy for high grade CIN might be a result of verification bias. The sensitivity looks high but is probably a spurious finding caused by the fact that most studies restricted excision mainly to women with a positive punch biopsy” [20].
			β“ There exists a wide range of physical diagnostic tests for FAI and/or labral pathology and little information on the diagnostic accuracy and validity. The methodological quality of the diagnostic accuracy studies is moderate to poor ” [23].
Results of quality assessment reported and discussed, and recommendations based on general unspecified quality items		12 (18%)	Assessed quality with Original QUADAS. Only included high quality studies based on a summary score (>9/14) “In conclusion, T2WI combined with DWI is superior to T2WI alone in the detection of prostate cancer. High-quality prospective studies regarding the combination of T2WI plus DWI in detecting prostate carcinoma still need to be conducted” [30].

^b^Quality was mentioned in one or more sections in the main text.

α Example of conclusion in a review with a meta-analysis.

β Example of conclusion in a review without a meta-analysis.

### Incorporation in the analysis

Twelve of the included reviews did not contain a meta-analysis. Four reviews [22-24,31] cited the poor quality of the identified studies as a reason for not conducting a meta-analysis, three [22-24] of which further factored the poor quality of studies in their conclusion. Other reasons for not conducting a meta-analysis were heterogeneity in test executions or study populations (n = 5) and not meeting inclusion criteria (n = 1); 2 reviews did not give an explanation.

Among the reviews with a meta-analysis (n = 53), nineteen (36%) incorporated quality in the analysis. Quality was incorporated in the analysis using meta-regression (n = 6), sensitivity analysis (n = 4), subgroup analysis (n = 2), both meta-regression and subgroup analysis (n = 2) or through unspecified methods, (n = 5). Eight found significant effects of quality on accuracy; in none of them these effects were factored in the conclusions.

### Incorporation in the discussion

Thirteen reviews (20%) only presented results of quality assessment, without further discussion; most of these (n = 12) contained a meta-analysis. In total, 47 reviews (72%) discussed the results of quality assessment but only 6 (9%) further linked these results to their conclusions.

Ten reviews without a meta-analysis discussed their results but only four [21-24] linked these results to their conclusions. Quality was discussed as a study limitation (n = 7), as a strength of the review (n = 2) and as potentially influencing the accuracy estimates (n = 1).

For the reviews with a meta-analysis, the results of the quality assessment were discussed 35 times in the discussion section, and twice in the results section. In the discussion section, quality was discussed as a study limitation (n = 21), as a strength of the study (n = 7), as a summary of results of the analysis (n = 11), and as potentially influencing the summary estimates of the review (n = 4). Eight studies discussed quality in more than one way. In the results section, quality was discussed as potentially influencing the summary estimates of the review (n = 1) and as strength of the review (n = 1). Twenty of the reviews that did not incorporate quality in their analysis (n = 30) discussed their results of quality assessment. They did so mostly as limitations in assessing the quality of included studies (n = 14, 70%).

### Incorporation in conclusions

In total, only 6 reviews (9%) incorporated the results of quality assessment in their conclusions in the main text of the review [20-24,32]. Most of which (n = 4) were reviews without a meta-analysis [21-24]. Three reviews cited poor quality as a reason for not conducting a meta-analysis [22-24].

Of these 6 reviews that incorporated quality in the conclusions, 3 were published in a journal with an impact factor above the median impact factor (3.1) of the included reviews. In addition, 2 reviews were imaging studies and 4 reviews evaluated tests belonging to the category ‘other’.

For the reviews with a meta-analysis, one acknowledged the limitations in assessing the quality of included studies, [32] and one other considered the potential effect of the quality item ‘verification bias’ on the test’s accuracy estimates [20]. These reviews did not highlight the quality of included studies (high or low quality) in the main text and had not performed any statistical analysis to investigate the effect of quality differences on pooled estimates.

Of these two reviews, one also incorporated results of quality assessment in the conclusion in the abstract [20]. The other review [32] encouraged authors in the conclusion of the main text to be cautious when interpreting the results of the review, because of the methodological limitations, but did not highlight this limitation in the conclusion of the abstract. An abstract that presents overly optimistic conclusions compared to the main text may lead to overinterpretation of the test’s accuracy results [33].

Twelve reviews made recommendations about the test in the main text, based on general unspecified quality items not linked to the results of quality assessment, and using phrases such as ‘high quality studies are needed’ or ‘large prospective studies are needed’. These were all reviews with a meta-analysis.

## Discussion

In a sample of 65 recently published diagnostic accuracy reviews of which 53 contained a meta-analysis, we found that almost all (92%) had assessed the methodological quality of included studies. Yet only 6 reviews (9%) considered results of quality assessment when drawing conclusions about the test’s performance. Three of these had decided not to perform a meta-analysis because of limitations in quality of the available evidence.

Whiting and colleagues [34] have previously reviewed existing quality assessment tools for diagnostic accuracy studies, two years after the introduction of the original QUADAS tool. They examined to what extent quality had been assessed and incorporated in diagnostic systematic reviews. Just about half of the 114 systematic reviews examined had assessed the methodological quality of included studies; 91 different quality assessment tools were identified. In contrast, only 5 different quality assessment tools could be identified in our study, with QUADAS being used in about 8 in 10 reviews assessed. This reinforces the existing evidence on the rapid uptake of QUADAS [12,13].

Whiting and colleagues observed that 11 reviews (10%) used study quality as a basis for recommendations for future research. Yet it was unclear if these recommendations were based on the quality as documented in the reviews. Recommendations for future research can also be based on aspects not necessarily investigated in the review. Our study showed that twelve reviews made recommendations about the test based on general unspecified quality items not linked to the results of quality assessment, using rather general phrases, such as ‘high quality studies are needed’ or ‘large prospective studies are needed’.

The specific reasons for not considering the assessments of quality of included studies in the overall findings of reviews are unclear. The absence of quality considerations could be partly explained by the parallel absence of clear recommendations on how to do so: guidance on how to incorporate quality into the conclusions of a review is scarce and vague.

Key guidance papers on reporting and evaluating systematic reviews, such as the Cochrane handbook [3,4,35], the statements on preferred reporting items for systematic reviews and meta-analyses (PRISMA) [36], on the assessment of multiple systematic reviews (AMSTAR) [37], and on the grading of recommendations assessment, development and evaluation (GRADE) [38,39] recommend that the methodological quality of included studies is discussed and factored into the overall findings of the review, but all of these fall more or less short on clearly explaining how to do so.

For instance, the Cochrane handbook for reviews of diagnostic accuracy studies [4,35] recommends that quality is assessed, included in the analysis, and used to generate recommendations for future research. It does not explicitly state how to discuss the results and incorporate the findings into the conclusions. The PRISMA guideline [36] is explicit in recommending that authors present the results of the risk of bias assessment and highlight, in the discussion section, any limitations encountered during risk of bias assessment. About the conclusion section, the recommendation in PRISMA is more vague; it advises authors to ‘provide a general interpretation of the results in the context of other evidence, and implications for future research’. AMSTAR [37] is a scoring system for evaluating the quality of a systematic review, rather than that of the studies included in such a review. One item it recommends, as a measure of the quality of a review, is whether the review used the quality of included studies in formulating conclusions (Item 8). GRADE [38,39] provides a framework for making evidence based recommendations by rating the quality of the evidence and grading the strength of recommendations. In this process risk of bias assessment is a key component. The strength of GRADE lies in providing guidance on how to make recommendations; it does not stipulate how risk of bias assessment can be incorporated in evidence synthesis.

Another aspect to be held responsible for the absence of quality considerations in the conclusions of systematic reviews may be the multidimensional nature of evaluations of risk of bias. Since there are multiple quality or risk of bias items to consider, review authors may find it difficult to select the most important quality items to assess, analyze, discuss and draw conclusions from. Some authors use a summary score, a quantitative estimate of quality items evaluated. However, the use of such simple summary scores is discouraged because they fail to consider differences in importance of quality items [40,41].

Poor reporting of relevant items in primary diagnostic accuracy studies, as stipulated by the Standards for Reporting of Diagnostic Accuracy initiative (STARD) [7], limits the assessments of quality of these studies. Authors may find it challenging to draw conclusions about the quality of the included studies and their impact on the test accuracy estimates when their assessments of quality or risk of bias are unclear. Many authors of reviews in our study discussed the challenges in assessing the quality of included studies as a review limitation.

Our study has one main limitation. Given that QUADAS-2 was recently introduced - just one year before the time of our search - and that uptake of novel methods takes time, we did not expect to find many articles utilizing the new version. This limited our evaluation of how results using QUADAS-2 are incorporated into the conclusions. Nonetheless, we anticipate that drawing conclusions from the multiple domains of risk of bias recommended by QUADAS-2 will still be challenging.

Although most reviews in our study did not consider quality in drawing conclusions, the ones that did show that it is possible to consider the strength of the evidence in making statements about a test’s performance based on a systematic review of test accuracy studies. If there is no quality evidence, one can refrain from meta-analysis, and make no firm statements about test performance. Alternatively, one can explicitly qualify the results from a meta-analysis of poor quality studies as evidence with limited credibility. If there are studies with and studies without deficiencies, one can limit the analysis to high quality studies, and add explicit statements to that extent to the conclusions. If there are studies with high risk of bias and studies at low risk, one can explore the effects of this variability on the summary estimates. If there are systematic effects, one could and should factor this finding into the conclusions. The dominant practice seems the worst possible scenario: to evaluate the quality of included studies without considering the findings from that exercise in drawing conclusions.

Guidance is needed in assisting authors to incorporate results of quality assessment in the conclusions. Such guidance should come from concerted actions of methodologists. It could be presented in the form of simple and practical online tutorials or tutorials published in scientific journals. Such tutorials could guide authors with examples on how to draw conclusions, especially in light of challenges such as the multiple domains of risk of bias recommended by QUADAS-2, when quality of included studies has no statistical effect on the pooled accuracy estimates, or when the risk of bias assessment is hampered by poor reporting of included studies, or when poor quality of studies precludes a meta-analysis.

## Conclusion

We found it disturbing that quality of the included evidence was evaluated in almost all diagnostic reviews, but that almost no authors had incorporated the results of quality assessment in the conclusions of their reviews. The practice of reporting systematic reviews of test accuracy should improve if readers not only want to be informed about the limitations in the available evidence, but also on the associated implications for the performance of the evaluated tests in clinical practice. Reviewers and readers of test accuracy reviews need to check that the results or limitations of quality assessment are incorporated in the abstract and conclusion of the review. Simply relying on the review results, without considering the quality of the underlying research, could lead to the uptake of poorly performing tests in practice and, consequently, to suboptimal patient management.

## Competing interests

No funding was received for this project. JR and PB were involved in the development of both the original and revised QUADAS tool. KGM and ML were involved in the development of the revised QUADAS tool.

## Authors’ contribution

Design of study: EO, WE, CN, LH, JG, JR, KGM, PB, ML. Data collection: EO, WE, CN, LH, JG, ML, PB. Data analysis: EO, ML. Data interpretation: EO, WE, CN, LH, JG, JR, KGM, PB, ML. Drafting of manuscript: EO, WE, CN, LH, JG, JR, KGM, PB, ML. Final approval of manuscript: EO, WE, CN, LH, JG, JR, KGM, PB, ML.

## Authors’ information

**EO:** PhD researcher, Department of Clinical Epidemiology, Biostatistics & Bioinformatics Academic Medical Center, University of Amsterdam, The Netherlands and Senior researcher, Centre for Evidence-based Health Care, Faculty of Medicine & Health Sciences, Stellenbosch University, South Africa

**WE:** PhD student, Department of Clinical Epidemiology, Biostatistics & Bioinformatics and Dutch Cochrane Center, Academic Medical Center, University of Amsterdam, The Netherlands

**CN:** PhD student, Julius Center for Health Sciences and Primary Care, University Medical, The Netherlands

**LH:** Senior epidemiologist, Department of Clinical Epidemiology, Biostatistics & Bioinformatics and Dutch Cochrane Center, Academic Medical Center, University of Amsterdam, The Netherlands

**JG:** Assistant professor, Julius Center for Health Sciences and Primary Care, University Medical Center Utrecht, The Netherlands

**JR:** Associate Professor, Julius Center for Health Sciences and Primary Care, University Medical Center Utrecht, The Netherlands

**KGM:** Professor of clinical epidemiology, Julius Center for Health Sciences and Primary Care, University Medical Center Utrecht, The Netherlands

**PB:** Professor of clinical epidemiology, Department of Clinical Epidemiology, Biostatistics & Bioinformatics, Academic Medical Center, University of Amsterdam, The Netherlands

**ML:** Assistant Professor, Department of Clinical Epidemiology, Biostatistics & Bioinformatics, Academic Medical Center, University of Amsterdam, The Netherlands

## Pre-publication history

The pre-publication history for this paper can be accessed here:

http://www.biomedcentral.com/1471-2288/14/33/prepub

## Supplementary Material

Additional file 1 Search strategy. Click here for file
