# Supplementary material for: Incorporating quality assessments of primary studies in the conclusions of diagnostic accuracy reviews: a cross-sectional study
Source: BMC Med Res Methodol. 2014 Mar 3;14:33. doi: 10.1186/1471-2288-14-33 (PMC3942773; doi:10.1186/1471-2288-14-33)
Supplement: Additional file 1 — Search strategy. [file 1471-2288-14-33-S1.pdf]

Additional File 1. Search strategy

1. systematic.mp. [mp=ti, ab, sh, hw, tn, ot, dm, mf, dv, kw]
2. limit 1 to "reviews (best balance of sensitivity and specificity)"
3. predict*.ti,ab.
4. test.ti,ab.
5. tests.ti,ab.
6. 4 or 5
7. 2 and 3 and 6
8. screen*.mp. [mp=ti, ab, sh, hw, tn, ot, dm, mf, dv, kw]
9. 2 and 8
10. monitoring.mp. [mp=ti, ab, sh, hw, tn, ot, dm, mf, dv, kw]
11. 2 and 10
12. "multiple tests".mp. [mp=ti, ab, sh, hw, tn, ot, dm, mf, dv, kw]
13. 2 and 12
14. "diagnostic test accuracy".mp. [mp=ti, ab, sh, hw, tn, ot, dm, mf, dv, kw]
15. DTA.ti,ab.
16. exp "sensitivity and specificity"/
17. specificit*.tw.
18. "false negative".tw.
19. accuracy.tw.
20. 14 or 15 or 16 or 17 or 18 or 19
21. 2 and 20
22. 7 or 9 or 11 or 13 or 21
23. limit 22 to (english language and yr="2012")
